# Validation of the femoral anteversion measurement method used in imageless navigation

**DOI:** 10.3109/10929088.2012.690230

**Published:** 2012-06-08

**Authors:** Glen A. Turley, Shahbaz M.Y. Ahmed, Mark A. Williams, Damian R. Griffin

**Affiliations:** 1 Product Evaluation Technologies Group, WMG, The University of Warwick, Coventry; 2 Trauma and Orthopaedic Surgery, Warwick Medical School, The University of Warwick, Coventry, United Kingdom

**Keywords:** Total hip arthroplasty, biomechanics, computer-assisted navigation, computed tomography, femoral anteversion, gait analysis

## Abstract

Total hip arthroplasty restores lost mobility to patients suffering from osteoarthritis and acute trauma. In recent years, navigated surgery has been used to control prosthetic component placement. Furthermore, there has been increasing research on what constitutes correct placement. This has resulted in the definition of a safe-zone for acetabular cup orientation. However, there is less definition with regard to femoral anteversion and how it should be measured. This study assesses the validity of the femoral anteversion measurement method used in imageless navigation, with particular attention to how the neutral rotation of the femur is defined. CT and gait analysis methodologies are used to validate the reference which defines this neutral rotation, i.e., the ankle epicondyle piriformis (AEP) plane. The findings of this study indicate that the posterior condylar axis is a reliable reference for defining the neutral rotation of the femur. In imageless navigation, when these landmarks are not accessible, the AEP plane provides a useful surrogate to the condylar axis, providing a reliable baseline for femoral anteversion measurement.

## Introduction

A successful total hip replacement normalizes the biomechanics of the hip joint, enabling a patient to regain mobility without pain or discomfort [1]. Normalization of hip joint biomechanics is dependent upon achieving joint stability and the ideal range of motion for a patient to fulfil their daily activities [2]. This is dependent upon achieving the correct prosthetic component orientation. A number of values have been recommended for the optimum orientation of the pelvic acetabular cup in order to achieve a stable hip joint without risk of dislocation [3–11]. Yoon et al. [12] resolved the inconsistencies associated with acetabular component positioning and found that it should be positioned with 41° cup inclination and 16° cup anteversion, using radiographic angles [13]. Due to the inter-dependence of acetabular cup anteversion and the version of the femoral stem, combined version values have been posed by adding recommended values for acetabular cup anteversion to those recommended for femoral version [14, 15]. Clinical recommendations for the amount of combined version vary between 25° and 60° [8, 16–18]. It has not only been difficult to define optimal component orientation; it has also been problematic to control prosthetic component orientation. Malchau et al. [19] analyzed the acetabular cup inclination and anteversion measurements of 1952 THA procedures. It was found that only 47% of patients had a cup orientation within both the defined cup inclination and anteversion boundaries. To improve prosthetic component positioning, computer-assisted navigation has been employed to improve both the accuracy and precision of acetabular component placement compared to the respective associated manual techniques. Many studies have demonstrated both improved accuracy and a reduction in outliers as a result of using computer assistance [20–24].

While many articles have been published concerning the validity and reliability of computer-assisted navigation with regard to the orientation of the pelvic acetabular cup, less has been written about femoral stem placement. This study aimed to assess the method used by an imageless navigation system to determine the neutral rotation of the femur from which the measurement of femoral anteversion is determined.

## Background

**Figure 1:**
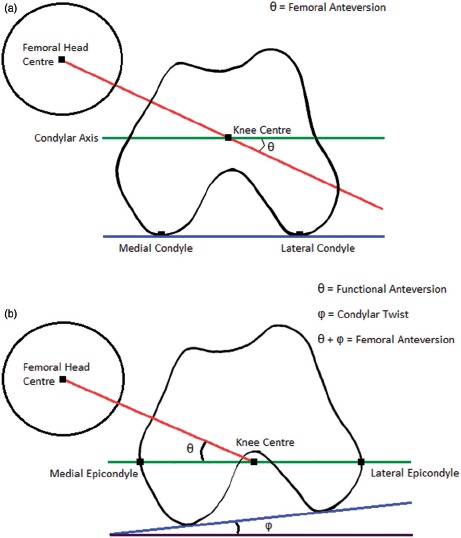
Measurement of femoral anteversion and the effect of condylar twist angle. (a) Measurement of femoral anteversion - angle between the condylar axis (green) and the femoral neck axis (red) [25]. (b) The condylar twist angle, caused by the posterior projection of the medial femoral condyle being greater than that of the lateral condyle, which externally rotates the transepicondylar axis of the femur [28].

Femoral anteversion has been defined by Murphy et al. [25] as the angle between the femoral neck axis and an axis parallel to the posterior aspect of the femoral condyles, measured in the transverse plane. This axis is known as the condylar axis and is shown in Figure 1a. The condylar axis is used to define the neutral rotation of the femur, and is therefore coincident with the coronal plane of the hip joint [26]. However, the femoral condyles are not accessible to permit assessment of whether the condylar axis is coincident with the coronal plane when a subject is posed in the anatomical neutral posture. As an alternative, Wu et al. [27] base the neutral rotation of the femur from the transepicon-dylar line in their non-orthogonal joint coordinate frame. The transepicondylar line is considered to be externally rotated relative to the coronal plane. This is due to the condylar twist angle caused by the posterior projection of the medial femoral condyle being greater than that of the lateral condyle, as shown in Figure lb [28].

**Figure 2:**
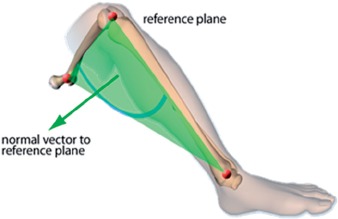
Ankle epicondyle piriformis (AEP) plane formed by the mid-point of the ankle malleoli, the mid-point of the femoral epicondyles, and the piriformis fossa. The normal vector to this plane is used instead of the condylar axis in imageless navigation to define the neutral rotation of the femur

In imageless navigation, the ankle epicondyle piriformis (AEP) plane is used instead of the condylar axis to define the neutral position of the femur. The AEP plane replicates the ‘figure-of-four’ axis used in non-navigated surgery as a reliable reference to the condylar axis [29]. The AEP plane is shown in Figure 2: It is formed by the mid-point of the ankle malleoli, the mid-point of the femoral epicondyles and the piriformis fossa. The normal vector to this plane along with the femoral mechanical axis defines the coronal plane of the femur. The mechanical axis is a line running in the positive direction from the mid-point of the femoral epicondyles to the hip joint center, defining the superior-inferior direction. A line perpendicular to the coronal plane forms the anterior-posterior axis, with the medial-lateral axis orthogonal to the other two axes. This study aimed to assess the reliability of the condylar axis for estimating the neutral rotation of the femur, and whether the normal to the AEP plane is coincident with the condylar axis. If both are true, then the basis on which femoral anteversion is measured in imageless navigation can be considered valid and reliable.

## Materials and methods

To assess whether the condylar axis accurately defines the neutral rotation of the femur, the angle between the pelvic medial-lateral axis and the femoral transepicondylar axis was measured in the transverse plane. Before performing the measurement, the condylar axis was aligned with the coronal plane in which the medial-lateral axis lies. This angle was measured on 10 subjects using a CT-based method. The angle was also measured in a separate cohort of 18 subjects using a gait analysis method, where it was not possible to align the condylar axis. The purpose of this was to assess the agreement between the two measurements: If agreement was found, then it could be inferred that when a subject is stood in the anatomical neutral posture the condylar axis lies in the coronal plane. Therefore, the method of Murphy et al. [25] can be used reliably to define the neutral rotation of the femur when the posterior aspect of the femoral condyles is accessible.

The gait analysis method was then used to assess whether the normal vector to the AEP plane was coincident with the condylar axis. The angle between the pelvic medial-lateral axis and the femoral medial-lateral axis was measured. This angle was measured in the transverse plane, providing a measure of the difference between the pelvic coronal plane and the femoral coronal plane in which the AEP normal vector lies. If minimal deviation was found to exist, then it could be inferred that the normal vector to the AEP plane can reliably define the neutral rotation of the femur. Consequently, the AEP plane can be used reliably in imageless navigation where the posterior aspect of the femoral condyles is not accessible.

### CT method

To measure, in the transverse plane, the angle between the pelvic medial-lateral axis and the femoral transepicondylar axis, CT scans were acquired of 10 subjects in the supine position. The subjects were all male and exhibited no evidence of osteoarthritis or abnormal morphology. Table II lists the age of each subject. The scans were acquired on a General Electric lightSpeed CT scanner with a slice thickness of 1.25 mm, encompassing the complete anatomy of the pelvis and femur. Each of the CT scans was segmented to produce three-dimensional (3D) models of the pelvis and femur. Each slice of every patient CT scan was imported into the ImageJ image-processing software (http://rsbweb.nih.gov/ij/) and segmented so that only matter with the same density as bone remained. Each slice was then cleaned, removing any non-bone material and filling gaps in the pelvic and femoral traces. This ensured maximum fidelity with regard to the bony landmarks of the femur and pelvis. Each cleaned image stack was then imported into the Simpleware ScanIP software package (Simpleware Ltd., Exeter, UK). A morphological smoothing filter set at one-pixel spacing was applied to smooth the inconsistencies between slices, and a 3D model mesh was then generated for the pelvic and femoral masks. These 3D models were then imported into the Rhino 4.0 NURBS modeling package (McNeel, Seattle, USA) for measurement.

The pelvic and femoral 3D models had to be aligned in order to be able to measure the angle between the pelvic medial-lateral axis and the femoral transepicondylar axis. The pelvis was aligned based on the landmarks of the transverse pelvic plane (TPP) [30]. The medial-lateral axis was defined as a line running parallel to the two anterior superior iliac spines (ASIS) running in the positive direction from left to right with the origin at the hip joint center. The hip joint center was defined as the center of a best-fit sphere of the femoral head. The transverse plane was defined as a plane containing the two ASIS and the mid-point of the two posterior superior iliac spines (PSIS). A line perpendicular to the transverse plane with the origin at the hip joint center defined the superior-inferior direction. The anterior-posterior axis was constructed orthogonal to the other two axes.

**Figure 3:**
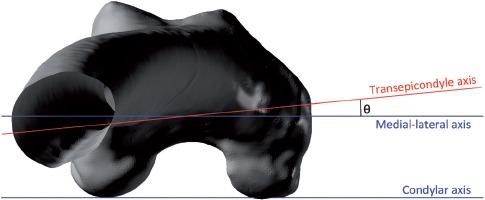
The angle between the medial-lateral and transepicondylar axes when viewed in the transverse plane.

The coordinate system of the femur was defined according to the standard defined by Murphy et al. [25]. The superior-inferior or mechanical axis was defined as running in the positive direction from the knee center to the hip joint center. The knee center was defined by the mid-point of the two femoral epicondyles. The coronal plane was defined as a plane containing the hip joint center and a line parallel to the posterior aspect of the femoral condyles located at the knee center. The anterior-posterior axis was constructed perpendicular to the coronal plane located at the hip joint center, and the medial-lateral axis was constructed orthogonal to the other two axes. The femoral 3D model was then aligned so that its axes were coincident with the coordinate frame of the pelvis. The angle in the transverse plane was then measured between the coronal medial-lateral axis and the transepicondylar axis formed as a line between the two femoral epicondyles, as shown in Figure 3.

### Gait analysis method

**Figure 4:**
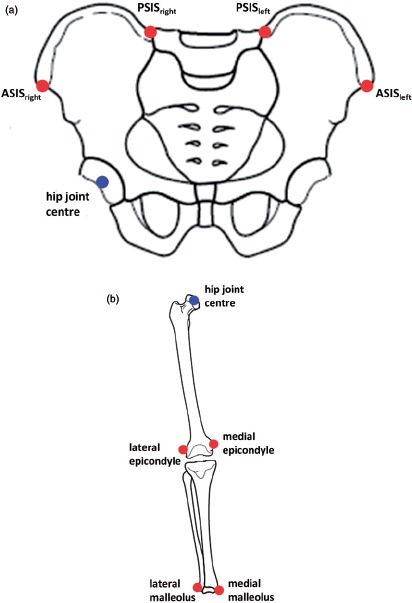
Gait analysis method marker positions, (a) Pelvic marker locations (in red), (b) Leg marker locations (in red).

The gait analysis method was used to measure two angles. First, the angle between the pelvic medial-lateral axis and the femoral transepicondylar axis in the transverse plane was measured. Secondly, the angle between the pelvic medial-lateral axis and the femoral medial-lateral axis was measured, again in the transverse plane. Eighteen subjects were recruited to perform three experiments. The subjects were all male and exhibited no evidence of osteoarthritis or abnormal morphology. Table III lists the age of each subject. Measurements were taken using a Vicon MX motion-capture system (Vicon, Oxford, UK) housed at the Gait Laboratory within the School of Engineering, University of Warwick. This system consisted of 12 infrared cameras located around the laboratory. The cameras tracked the 3D coordinate locations of passive marker spheres placed on the subject. The marker spheres were placed on the subject in the following locations: the medial and lateral ankle malleoli, the medial and lateral femoral epicondyles, the pelvic right and left ASIS, and the pelvic right and left PSIS. This configuration is shown in Figure 4.

The first experiment determined the center of the hip joint. This landmark was estimated based on the motion of the femur relative to the pelvis. It was required to be determined because the landmark of the piriformis fossa was not accessible. Therefore, to form the AEP plane an alternative proximal femoral landmark was used as a surrogate, namely the hip joint center. To begin the experiment, the subject was asked to stand, positioning himself so that the centers of his knees were directly below the centers of his hips, defining the neutral posture [31, 32]. The subject was then asked to flex, extend, abduct and adduct his femur in the star-arc motion, as recommended by Camomilla et al. [33]. Once this was completed, the marker trajectories were used to calculate the hip joint center using the bias-compensated least squares estimate of center of rotation developed by Halvorsen [34]. The hip joint center was calculated relative to the local pelvic coordinate frame based on the TPP. The mathematical formulae used to determine the hip joint center in the first experiment are shown in Appendix 1.

In the second experiment, the angle between the pelvic medial-lateral axis and the transepicondylar axis was measured. Similar to the first experiment, the subject was asked to stand so that the centers of his knees were directly below the centers of his hips, defining the neutral posture [31, 32]. The pose was held for a period of 10 seconds. Once complete, the marker positions of the medial and lateral femoral epicondyles were used to construct the transepicondylar axis. The angle in the transverse plane was then calculated between this axis and the medial-lateral axis of the pelvic coordinate frame. The mathematical formulae used to calculate this angle are shown in Appendix 2.

In the final experiment, the angle between the pelvic medial-lateral axis and the femoral medial-lateral axis was measured. Again, to start the experiment the subject was asked to stand in the neutral posture. The subject was then asked to flex his hip to approximately 65° and his knee to 90°, ensuring minimal leg abduction or adduction. Once this was completed, the AEP plane was calculated using the estimated hip joint center, the mid-point of the two femoral epicondyles, and the mid-point of the two ankle malleoli. The AEP plane normal was determined and used to construct the femoral coordinate frame. The angle between the pelvic medial-lateral axis and the femoral medial-lateral axis was then measured. This measurement was taken in the transverse plane where the normal vector to the AEP plane is coincident with the femoral medial-lateral axis. Measurement of the minimum angle between the two axes was recorded, as well as the angular deviation at 25°, 45° and 65° of hip flexion. The mathematical formulae used to construct the AEP plane and calculate this angle are shown in Appendix 3.

### Statistical analysis

To assess whether the condylar axis lies in the coronal plane, thus defining the neutral position of the femur, the findings of Yoshioka and Cooke [28] were used for reference. Their study found that, on average, the angle in the transverse plane between the condylar axis and the transepicondylar axis was 5.7° *﹛a = 2.2°)*. Based on these findings, it was determined that the transepicondylar axis in both the CT and gait analysis experiments should be externally rotated by an average of 5.7° when measured from the pelvic medial-lateral axis. To assess the agreement between the experimental means, two statistical tests were performed. First, a t-test using the *a* value determined by Yoshioka and Cooke [28] with a sample size of 28 subjects was used. This test had an 80% power to detect a 2.5° difference between the experiment means. A second test measured the effect size between the two experimental methods. The effect size was determined using the percentage variance in scores (PV) [35]. The percentage variance calculates the variation between the means of the dependent variable measured in the two experiments as a proportion of the total variation (Equation 1). The calculated PV was used to classify the effect size of the difference in means between the two experimental methods, as shown in Table I [35].


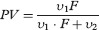


**Table I:** Effect size.

Effect size	PV
Small	0.01
Small/medium	0.05
Medium	0.1
Large	0.25

To assess whether the normal vector to the AEP plane lies in the coronal plane, a mean angle between the medial-lateral axes of the pelvis and femur was defined. This angle should be within 2.5° with a *a = 2.5°* when measured in the transverse plane. If the results of this experiment met the criteria, then the normal vector to the AEP plane could be considered to lie in the coronal plane and could be used as a basis from which to measure the anteversion of the femoral neck.

## Results

Using the CT-based method, the angle in the transverse plane between the pelvic medial-lateral axis and the femoral transepicondylar axis was measured to be, on average, 6.61° externally rotated (<r = 2.43°). The same angle, using the gait analysis method, was measured to be, on average, 4.12° externally rotated (<x=7.69°). The results for the CT and gait analysis methods are shown in Tables II and III, respectively. The t-test showed that there was no significant difference between the sample means of the two experimental methods (p-value = 0.332). The size of the effect between the sample means of the two experimental methods was between small and small/medium (PV = 0.036).

**Table II:** CT method: Angle between the condylar axis and transepicondylar axis measured in the transverse plane (negative value = external rotation).

Subject	Age	Angle
1	53	−6.87°
2	74	−5.26°
3	49	−8.76°
4	65	−1.40°
5	80	−5.73°
6	81	−6.16°
7	63	−5.51°
8	52	−10.21°
9	57	−8.16°
10	78	−7.99°

μ	65.2	−6.61°
σ	12.3	2.43°

**Table III:** Gait analysis method: Angle between the pelvic medial-lateral axis and the transepicondylar axis measured in the transverse plane (negative value=external rotation).

Subject	Age	Angle
1	24	−8.42°
2	34	−22.38°
3	38	6.16°
4	29	−13.92°
5	33	−8.49°
6	41	−10.51°
7	42	−0.81°
8	29	9.18°
9	27	−4.17°
10	36	2.54°
11	30	−0.66°
12	25	4.73°
13	26	−7.22°
14	25	−6.98°
15	24	−7.37°
16	42	−5.82°
17	34	1.28°
18	28	−1.25°

μ	31.5	−4.12°
σ	6.2	7.69°

As determined using the gait analysis method, the measurements in the transverse plane between the pelvic medial-lateral axis and the femoral medial-lateral axis are shown in Table IV. The mean difference between the two axes was 0.38° with a cr=1.06°. This met the criteria for agreement defined earlier in the study. Table IV also shows that the angle between the two axes can vary during the movement cycle. The hip flexion angles of 25° and 45° had better agreement between the axes and were less variable than a hip flexion angle of 65°. There was no correlation with regard to hip flexion angle and agreement of the AEP normal vector with the coronal plane.

**Table IV:** Gait analysis method: Angle between the pelvic and femoral medial-lateral axes measured in the transverse plane.

Subject	Minimum angle	25% flexion	45% flexion	65% flexion
1	0.002°	0.63°	5.63°	7.08°
2	0.001°	0.06°	2.35°	5.95°
3	−0.003°	2.96°	3.10°	0.74°
4	0.003°	2.91°	2.20°	3.40°
5	0.000°	2.79°	2.95°	1.65°
6	3.936°	6.65°	4.99°	5.59°
7	−0.001°	3.93°	5.10°	7.74°
8	−0.019°	3.53°	0.22°	2.58°
9	0.001°	1.24°	0.03°	1.56°
10	0.672°	4.91°	1.08°	3.08°
11	−0.152°	3.38°	2.98°	3.49°
12	0.001°	4.52°	2.63°	4.44°
13	0.003°	1.65°	2.89°	0.33°
14	−0.001°	1.38°	1.97°	2.97°
15	−0.005	1.60°	2.52°	2.50°
16	−0.003	0.82°	2.95°	6.60°
17	0.029	1.99°	2.83°	1.36°
18	2.357	2.97°	2.77°	5.67°

μ	0.379	2.66°	2.73°	3.71°
σ	1.056	1.68°	1.47°	2.27°

## Discussion

**Figure 5:**
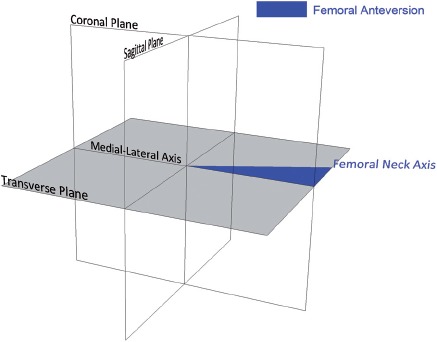
Anatomical femoral version, defined as the angle between the medial-lateral axis and the femoral neck axis measured in the transverse plane.

The measurement of femoral anteversion is congruent to the definition of anatomical anteversion presented by Murray [13], as shown in Figure 5. This angle uses as its basis the anatomical medial-lateral axis. The angle between this axis and the femoral neck axis, measured in the transverse plane, is used to define the amount of femoral anteversion. Therefore, it is critical that this medial-lateral axis is reliably defined and lies in the coronal plane when the subject is posed in the anatomical neutral position. This provides a valid start point from which hip joint range of motion can be calculated [36–38]. Murphy et al. [25] use the condylar axis to define the neutral rotation of the femur, and this has been accepted as standard [39, 40]. If this is true, then the condylar axis would lie in the coronal plane when a subject is stood in the neutral posture and is congruent to the medial-lateral axis when viewed in the transverse plane.

The null hypothesis for this study was that the condylar axis can reliably determine the neutral rotation of the femur when a person is posed in the anatomical neutral posture, upright and erect on both legs with the knee centers located below the hip joint centers [31, 32]. To determine whether this null hypothesis was true, the angle between the transepicondylar axis and the medial-lateral axis was measured in two scenarios. In the first scenario, the condylar axis could be determined and aligned with the pelvic medial-lateral axis. In the second scenario, the condylar axis was not accessible, but the subject was able to pose in the anatomical neutral posture. No significant difference was found between the two measurements, and both means were within 2° of the mean condylar twist angle measured by Yoshioka and Cooke [28]. This test supports the null hypothesis. The second test calculated the effect size between the two measurement methods. The effect size was found to lie in the small to small/medium range using the criteria set out in Table I. Even when the measurements made by Yoshioka & Cooke were pooled with the CT measurements and compared to the gait analysis measurements, the effect size still falls within this range (PV = 0.029). Therefore, this measurement does not provide complete support to the null hypothesis. However, any difference in the alignment between the condylar axis and the coronal plane when a subject is stood in the anatomical neutral posture is likely to be less than 3.5° at a 95% confidence level.

The second factor to be validated in this study was whether the normal vector to the AEP plane can be used to construct a femoral coordinate frame which accurately defines the neutral rotation of the femur. The results of this study have shown that the medial-lateral axes of the femoral and pelvic coordinate frames align extremely closely with a mean deviation of 0.38°. This validates the concept that when the posterior aspects of the femoral condyles are not available to construct the condylar axis, the AEP plane can be used to construct a coordinate frame which accurately defines the neutral rotation of the femur. Therefore, the AEP plane can be used to construct a reliable medial-lateral axis from which to measure femoral anteversion, similar to the ‘figure-of-four’ axis. There was evidence that the orientation of this axis varied throughout the movement cycle, and this variation had no correlation with hip flexion. It is hypothesized that this variation could be associated with tibial varus/valgus, although this was not measured in the study. Further work needs to be done to establish how to define a consistent AEP plane which can be used in imageless navigation.

There were a number of experimental assumptions which could affect the validity of the study findings. The first was associated with the reference frame used to construct the pelvic coordinate frame. Imageless navigation uses the anterior pelvic plane (APP), while this study used the reference points of the transverse pelvic plane (TPP) [37]. However, the difference in coordinate frame alignment does not affect the measurement result. This is because the medial-lateral axis of both the APP and TPP frames runs in the positive direction from the left ASIS to the right ASIS. Therefore, the measurement between the pelvic and femoral medial-lateral axes in the transverse plane is unaffected by pelvic plane definition. Secondly, in the gait analysis method, the landmark of the piriformis fossa was not accessible for use in the construction of the AEP plane. Therefore, the hip joint center was used as a surrogate for this landmark. To test the effect of this assumption, 3D models of a femur, tibia and fibula were imported into the Rhino 4.0 NURBS modeling package for measurement. The tibia and fibula were positioned to simulate 90° knee flexion. The difference in medial-lateral axis definition when using the hip joint center instead of the piriformis fossa was measured to be 0.15° in the transverse plane. Therefore, this supports the use of the hip joint center as a useful surrogate in the construction of the AEP plane.

This study assessed the validity of using the posterior aspects of the femoral condyles as a reliable reference for defining the neutral rotation of the femur. The measurements supported this assessment, although there was some variation. Also, it has been found that in imageless navigation the AEP plane can be used to define the neutral rotation of the femur. This provides a reliable basis from which to measure femoral anteversion.

## Appendix 1: Calculation of hip joint center

Landmark definitions:

ASIS: Anterior superior iliac spine.

PSIS: Posterior superior iliac spine.

LEPI: Lateral femoral epicondyle.

MEPI: Medial femoral epicondyle.

LMAL: Lateral ankle malleolus.

MMAL: Medial ankle malleolus.

*x*-axis: medial-lateral axis.

*y*-axis: anterior-posterior axis.

*z*-axis: superior-inferior axis.

*A11 calculations are for a left hip joint

Step 1: Translate all marker trajectories m,- with the PSIS_mid_ as the origin.

Step 2a: Pelvic x-axis.

Step 2b: Pelvic z-axis.

Step 2c: Pelvic _y-axis.

Step 3: Form pelvic body segment rotation matrix.

Step 4a: Translate all marker trajectories m,- with the ASIS_mid_ as the origin.

Step 4b: Transform LEPI and MEPI marker trajectories into the pelvic body segment coordinate frame.

Step 5: Calculate initial estimate of hip joint center using LEPI and MEPI marker trajectories [34].

Step 6: Compute correction term Δ^b and calculate hip_new_, repeat until convergence [34].

## Appendix 2: Stand experiment

hiPcenter = hip_new_

Pelvic medial-lateral axis *(y_x_)= [1 0 0]^T^*

Step 1: Translate all marker trajectories m,- with the hip_center_ as the origin.

Step 2: Calculate knee center.

Step 3a: Femoral z-axis.

Step 3b: Femoral _y-axis.

Step 3c: Femoral x-axis.

Step 4: Calculate angle in transverse plane between pelvis medial-lateral axis *(y_x_)*and transe-picondylar axis.

## Appendix 3: AEP experiment

Step 1: Translate all marker trajectories m,- with the hip_center_ as the origin.

Step 2: Calculate knee center.

Step 3: Calculate ankle center.

Step 4a: Femoral z-axis.

Step 4b: AEP vector.

Step 4c: Femoral v-axis.

Step 4d: Femoral x-axis.

Step 5: Calculate angle in transverse plane between pelvic *(v_x_)*and femoral *(vjx)*medial-lateral axes.

## References

[1] Sakai T, Sugano N, Nishii T, Haraguchi K, Ochi T, Ohzono K (2000). Optimizing femoral anteversion and offset after total hip arthroplasty, using a modular femoral neck system: An experimental study. J Orthop Sci.

[2] Duwelius PJ, Hartzband MA, Burkhart R, Carnahan C, Blair S, Wu YX, Grunkemeier GL (2010). Clinical results of a modular neck hip system: Hitting the bulls-eye more accurately. Am J Orthop.

[3] Biedermann R, Tonin A, Krismer M, Rachbauer F, Eibl G, Stockl B (2005). Reducing the risk of dislocation after total hip arthroplasty: The effect of orientation of the acetabular component. J Bone Joint Surg Br.

[4] Dorr LD, Wan Z (1998). Causes of and treatment protocol for instability of total hip replacement. Clin Orthop Relat Res.

[5] Lewinnek GE, Lewis JL, Tarr R, Compere CL, Zimmerman JR (1978). Dislocations after total hip-replacement arthroplasties. J Bone Joint Surg Am.

[6] Masaoka T, Yamamoto K, Shishido T, Katori Y, Mizoue T, Shirasu H, Nunoda D (2006). Study of hip joint dislocation after total hip arthroplasty. Int Orthop.

[7] McCollum DE, Gray WJ (1990). Dislocation after total hip arthroplasty: causes and prevention. Clin Orthop Relat Res.

[8] Sakai T, Sugano N, Ohzono K, Nishii T, Haraguchi K, Yoshikawa H (2002). Femoral anteversion, femoral offset, and abductor lever arm after total hip arthroplasty using a modular femoral neck system. J Orthop Sci.

[9] Seki M, Yuasa N, Ohkuni K (1998). Analysis of optimal range of socket orientations in total hip arthroplasty with use of computer-aided design simulation. J Orthop Res.

[10] Widmer KH, Zurfluh B (2004). Compliant positioning of total hip components for optimal range of motion. J Orthop Res.

[11] Yoshimine F (2005). The influence of the oscillation angle and the neck anteversion of the prosthesis on the cup safe-zone that fulfills the criteria for range of motion in total hip replace ments. The required oscillation angle for an acceptable cup safe-zone. J Biomech.

[12] Yoon YS, Hodgson AJ, Tonetti J, Masri BA, Duncan CP (2008). Resolving inconsistencies in defining the target orientation for the acetabular cup angles in total hip arthroplasty. Clin Biomech.

[13] Murray DW (1993). The definition and measurement of acetabular orientation. J Bone Joint Surg Br.

[14] Malik A, Maheshwari A, Dorr LD (2007). Impingement with total hip replacement. J Bone Joint Surg Am.

[15] Widmer KH (2007). Containment versus impingement: Finding a compromise for cup placement in total hip arthroplasty. Int Orthop.

[16] Dorr LD, Malik A, Dastane M, Wan Z (2009). Combined anteversion technique for total hip arthroplasty. Clin Orthop Relat Res.

[17] Jolles BM, Zangger P, Leyvraz PF (2002). Factors predisposing to dislocation after primary total hip arthroplasty. J Arthroplasty.

[18] Ranawat CS, Maynard MJ (1991). Modern technique of cemented total hip arthroplasty. Tech Orthop.

[19] Malchau H, Callanan M, Bragdon C, Zurakowski D, Jarrett B, Rubash H (2001). An analysis of cup positioning in total hip arthroplasty: Quality improvement by use of a local joint registry. J Bone Joint Surg Br.

[20] Honl M, Schwieger K, Salineros M, Jacobs J, Morlock M, Wimmer M (2006). Orientation of the acetabular component: A comparison of five navigation systems with conventional surgical technique. J Bone Joint Surg Br.

[21] Kalteis T, Handel M, Bathis H, Perlick L, Tingart M, Grifka J (2006). Imageless navigation for insertion of the acetabular component in total hip arthroplasty: Is it as accurate as CT-based navigation?. J Bone Joint Surg Br.

[22] Kelley TC, Swank ML (2009). Role of navigation in total hip arthroplasty. J Bone Joint Surg Am.

[23] Parratte S, Argenson JNA (2007). Validation and usefulness of a computer-assisted cup-positioning system in total hip arthro plasty: A prospective, randomized, controlled study. J Bone Joint Surg Am.

[24] Sugano N, Nishii T, Miki H, Yoshikawa H, Sato Y, Tamura S (2007). Mid-term results of cementless total hip replacement using a ceramic-on-ceramic bearing with and without com puter navigation. J Bone Joint Surg Br.

[25] Murphy SB, Simon SR, Kijewski PK, Wilkinson RH, Griscom NT (1987). Femoral anteversion. J Bone Joint Surg Am.

[26] Maruyama M, Feinberg JR, Capello WN, D'Antonio JA (2001). Morphologic features of the acetabulum and femur: Anteversion angle and implant positioning. Clin Orthop Relat Res.

[27] Wu G, Siegler S, Allard P, Kirtley C, Leardini A, Rosenbaum D, Whittle M, D'Lima DD, Cristofolini L, Witte H (2002). ISB recommendation on definitions of joint coordinate system of various joints for the reporting of human joint motion. Part I: Ankle, hip, and spine. J Biomech.

[28] Yoshioka Y, Cooke TDV (1987). Femoral anteversion: Assessment based on function axes. J Orthop Res.

[29] Mayr E, Thaler M, Williams A, Moctezuma De La Barrera J, Krismer M, Nogler M (2007). The figure-of-four axis as a reference to determine stem rotation in hip arthroplasty. What does it really measure? A cadaver study. Acta Orthop.

[30] Dandachli W, Richards R, Sauret V, Cobb JP (2006). The transverse pelvic plane: A new and practical reference frame for hip arthroplasty. Comput Aided Surg.

[31] Luttgens K, Wells KF (1982). Kinesiology: Scientific Basis of Human Movement.

[32] Rowley DI, Dent JA (1997). The Musculoskeletal System: Core Topics in the New Curriculum. Hodder Arnold.

[33] Camomilla V, Cereatti A, Vannozzi G, Cappozzo A (2006). An optimized protocol for hip joint centre determination using the functional method. J Biomech.

[34] Halvorsen K (2003). Bias compensated least squares estimate of the center of rotation. J Biomech.

[35] Murphy KR, Myors B, Wolach AH (2008). Statistical Power Analysis: A Simple and General Model for Traditional and Modern Hypothesis Tests.

[36] Ko BH, Yoon YS (2008). Optimal orientation of implanted components in total hip arthroplasty with polyethylene on metal articulation. Clin Biomech.

[37] Turley GA, Ahmed SMY, Williams MA, Griffin DR (2011). Establishing a range of motion boundary for total hip arthroplasty. Proc Inst Mech Eng H.

[38] Yoshimine F, Ginbayashi K (2002). A mathematical formula to calculate the theoretical range of motion for total hip replacement. J Biomech.

[39] Kubiak-Langer M, Tannast M, Murphy SB, Siebenrock KA, Langlotz F (2007). Range of motion in anterior femoroacetabular impingement. Clin Orthop Relat Res.

[40] Tannast M, Kubiak-Langer M, Langlotz F, Puls M, Murphy SB, Siebenrock KA (2007). Non-invasive three-dimensional assess ment of femoroacetabular impingement. J Orthop Res.

